# The challenge of breeding for reduced off-flavor in faba bean ingredients

**DOI:** 10.3389/fpls.2023.1286803

**Published:** 2023-10-30

**Authors:** Antonio Lippolis, Wibke S. U. Roland, Ornela Bocova, Laurice Pouvreau, Luisa M. Trindade

**Affiliations:** ^1^Plant Breeding, Wageningen University & Research, Wageningen, Netherlands; ^2^Wageningen Food & Biobased Research, Wageningen University & Research, Wageningen, Netherlands

**Keywords:** plant based, off-flavors, faba bean, breeding, tannins, saponins, lypoxygenase

## Abstract

The growing interest in plant protein sources, such as pulses, is driven by the necessity for sustainable food production and climate change mitigation strategies. Faba bean (*Vicia faba L*.) is a promising protein crop for temperate climates, owing to its remarkable yield potential (up to 8 tonnes ha^−1^ in favourable growing conditions) and high protein content (~29% dry matter basis). Nevertheless, the adoption of faba bean protein in plant-based products that aim to resemble animal-derived counterparts is hindered by its distinctive taste and aroma, regarded as “off-flavors”. In this review, we propose to introduce off-flavor as a trait in breeding programs by identifying molecules involved in sensory perception and defining key breeding targets. We discuss the role of lipid oxidation in producing volatile and non-volatile compounds responsible for the beany aroma and bitter taste, respectively. We further investigate the contribution of saponin, tannin, and other polyphenols to bitterness and astringency. To develop faba bean varieties with diminished off-flavors, we suggest targeting genes to reduce lipid oxidation, such as *lipoxygenases* (*lox*) and *fatty acid desaturases* (*fad*), and genes involved in phenylpropanoid and saponin biosynthesis, such as *zero-tannin (zt)*, *chalcone isomerase (chi)*, *chalcone synthase (chs*), *β-amyrin (bas1)*. Additionally, we address potential challenges, including the need for high-throughput phenotyping and possible limitations that could arise during the genetic improvement process. The breeding approach can facilitate the use of faba bean protein in plant-based food such as meat and dairy analogues more extensively, fostering a transition toward more sustainable and climate-resilient diets.

## 1 Introduction

Natural resources depletion and climate change exacerbate the challenge to meet the future food demand in view of anticipated population growth by 2050 (Calicioglu et al., 2019). In the past decades, rising incomes and urbanization have driven the increased consumption of animal proteins. Animal-based products contribute significantly to environmental pressures, including land and water use, greenhouse gas (GHG) emissions, acidification and eutrophication potential. In the depicted scenario, the transition from meat-intensive diets towards plant protein-based diets (“protein transition”) is crucial to meet the climate change mitigation targets and ensure food security (D’Odorico et al., 2018).

The European food market has already witnessed a burgeoning trajectory of plant-based food retail sales, especially meat and dairy analogues (Aschemann-Witzel et al., 2021). Also known as replacers or alternatives, analogues are plant-based processed foods designed to resemble animal products in appearance, texture and taste (Fresán et al., 2019; Kyriakopoulou et al., 2019). At present, soybean, wheat, and pea-based products dominate the market for meat analogues, while soybean and almond-based products do for dairy analogues (Nawaz et al., 2022). A more recent area of development involves the use of soy and wheat protein in fish analogues (Nowacka et al., 2023). Environmental concerns around soy production in South America (e.g., deforestation, land-use change, transport-associated emissions) and increased nitrogen emissions have led to consumer and policy-maker interest in alternative pulse-based proteins (Tsolakis et al., 2019). Pulses are the dried seeds of certain legumes (e.g., pea, chickpea, lentil, lupin, faba bean, and diverse other dry beans), thus excluding those that are used as vegetables (e.g., fresh peas, or green beans) or for oil extraction (e.g., soybean and peanut) (Iriti and Varoni, 2017). Among pulses, the use of protein-rich ingredients from faba bean (*Vicia faba L.*) (i.e., flours, concentrates, isolates) is still minor (Multari et al., 2015).

**Figure 1:**
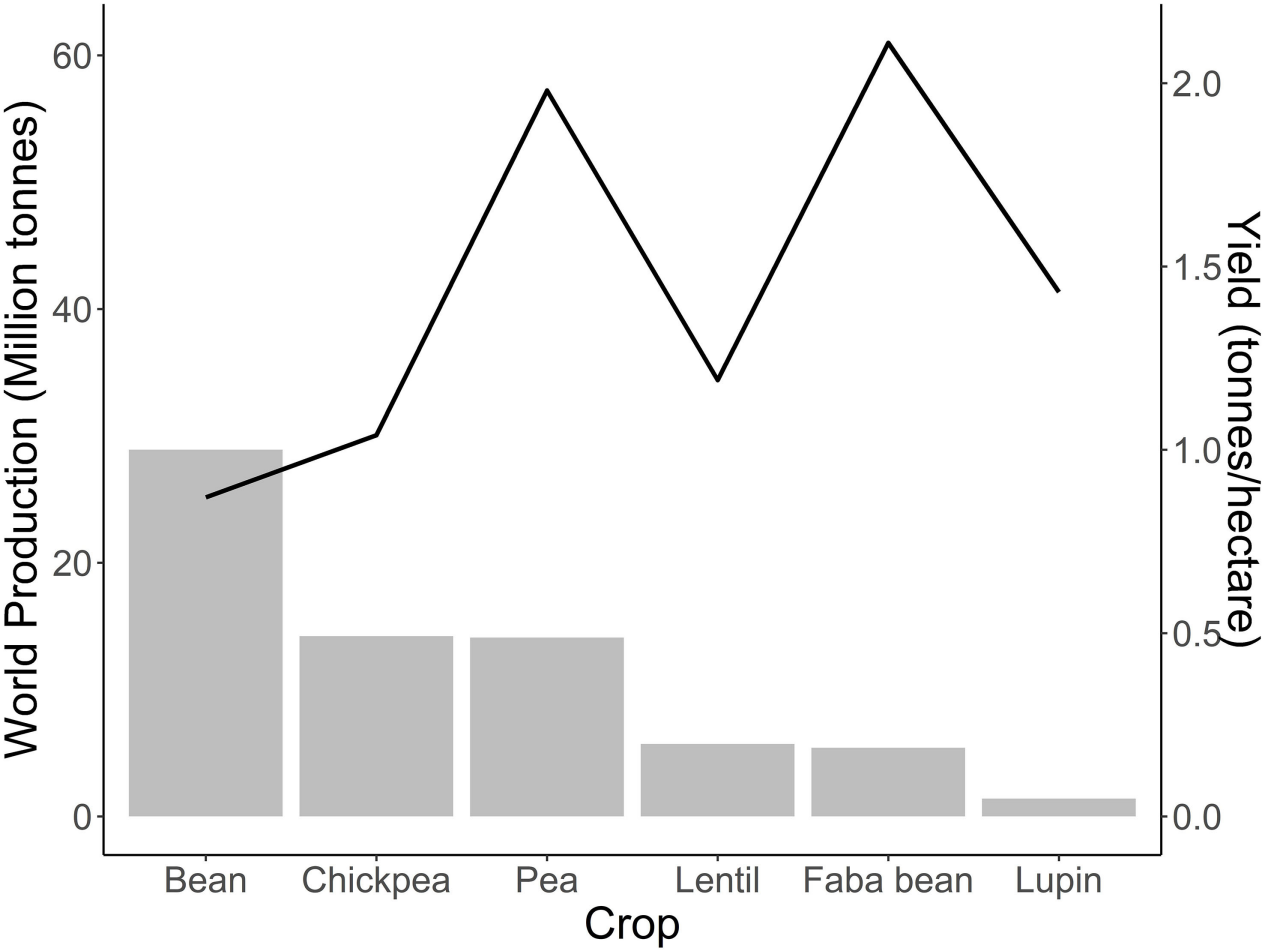
World production and yield of major pulses. Production in million tonnes of bean, chickpea, faba bean, lentil, lupin and pea for the year 2021. The trendline highlights the average yield of these crops as tonnes ha^−1^. The yield value reported was calculated by dividing world production by the total harvested area using data of 2021. Source: FAOSTAT, 2021.

Faba bean is in the spotlight for its high yield potential. The yield of superior cultivars adapted to specific environments and grown with appropriate husbandry can reach up to 8 to 9 tonnes ha−1 in favourable European climates (Metayer, 2004). However, less than half of the production potential is usually achieved due to biotic and abiotic stress, as well as inadequate agronomic management (Metayer, 2004). In Europe, the difference in actual yield between countries is remarkable. Yield ranges from an average of 3.9 t ha−1 in Germany and UK to 1.4 t ha−1 in the Mediterranean-type environments, such as Spain (Mínguez and Rubiales, 2021). Faba bean ranks behind other pulses such as lentil, cowpea, field pea, chickpea, and common bean in global harvested area and production, being superior only to lupin (FAOSTAT, 2021) (**Figure 1**). However, the high yield and protein content makes faba bean superior to most other pulses in terms of protein yield per unit of land area (protein ha−1).

*Vicia faba* represents one of the cheapest sources of proteins and carbohydrates. Carbohydrate content accounts for 50 to 68% (Punia et al., 2019), while protein content ranges from 20 to 40% (Dhull et al., 2022) of its seed composition, with an average of 29%. Contrarily, the oil content is low and ranges from 0.5 to 2%. Besides for high protein content, this crop is prized in the food industry because is locally grown and processed in Europe. Contrarily to imported soybean, faba bean supports “clean label” product (Asioli et al., 2017). Moreover, its seeds already have a long history of use in food worldwide (Zong et al., 2019), facilitating consumer acceptance of new products. This pulse is a healthy food source, containing potassium, calcium, magnesium, iron, and zinc, dietary fibres, phenolic compounds, 3,4-dihydroxyphenylalanine (L-DOPA), and γ-aminobutyric acid (GABA) (L’Hocine et al., 2020). Faba bean offers several agronomic and ecological advantages to growers, such as adaptability to a wide range of climates and soils, and a particularly high nitrogen-fixing ability via Rhizobium bacteria symbiosis (60-250 kg ha−1 per year) (Torres et al., 2011; Maalouf et al., 2018). Therefore, faba bean is an attractive option for both farmers and the food industry. To support the foreseen increase of its market share in the next years (Multari et al., 2015), it is necessary to develop and commercialise varieties with enhanced quality features that could facilitate the use of its protein fraction in meat or dairy analogues. Optimising the quality of the seeds for such food applications requires a synergistic approach between plant breeding and food technology research. In this way legume breeders will be guided by food technologists to the quality requirements of highly processed food including protein functionality (i.e., solubility, gelling, emulsifying or foaming property, etc.) and flavor. At the moment, the typical faba-bean-like flavor hampers consumer acceptance and is negatively perceived as “off-flavor” in meat and dairy analogues.

In this comprehensive review, we address the challenges associated with improving flavor features in faba bean, emphasising the need for specialised breeding programs tailored to ameliorate traits relevant to the modern food industry. The review will first provide a brief introduction on the cultivation, food uses, and breeding history of this crop. It will subsequently explore the molecules likely involved in flavor perception, elucidate the primary biosynthetic pathways controlling the production of these compounds, and identify the respective key regulators that can be targeted with breeding. Additionally, we will provide a perspective on breeding strategies aimed at mitigating off-flavors, discussing challenges such as high-throughput phenotyping and potential drawbacks in the process.

### 1.1 Traditional food uses and new trends of consumption

Faba bean is one of the earliest domesticated crops, but knowledge about its origin and pre-domestication distribution has been scarce and debatable (Duc et al., 2010). Cubero (1974) reported the Near East as the centre of origin. From this area, *Vicia faba* cultivation spread following human migrations to the Mediterranean basin, the Nile Valley, and Central and East Asia, all becoming regions of historical cultivation of this crop (Cubero, 1973). In area where the consumption of faba bean is a customary practice there are well known typical dishes based on faba bean such as ‘Ful Medames’ (Egypt), ‘Shahan ful’ (Ethiopia), ‘Ful bi’l-kammun’ (Tunisia), Bissara (Morocco), Bakla (Turkey), ‘Macco’, and ‘Puré di fave e cicorie’(Italy) (Pasqualone et al., 2020). In many Western nations (e.g., central-northern Europe and USA), faba bean is mainly used in animal feed. Overall, only about 15% of the total crop production targets human consumption, contributing to the perception that faba bean is an underutilised protein source. Despite being a healthy food, faba bean contains antinutritional factors, such us tannins, lectins, trypsin inhibitors that can reduce protein digestibility and negatively impact the bioavailability of minerals and polysaccharides (Kim et al., 2015; Rahate et al., 2021). Additionally, vicine and convicine cause favism, in individuals who have a hereditary loss of the enzyme glucose-6-phosphate dehydrogenase (Luzzatto and Arese, 2018).

The interest of the food industry in utilising faba bean protein fractions as food ingredients has been growing. This is witnessed by increased literature on the incorporation of faba bean flour and protein-rich fractions in formulations for bread, pasta, snack, infant formulas, plant-based meat analogues, etc. By replacing 30% of wheat flour with faba bean flour, bread formulations have shown an increase in protein content from 11.6% to 16.5% of dry matter (Coda et al., 2017). The partial substitution of wheat with faba bean ingredients in pasta has also been suggested to enhance nutritional properties for better health outcomes (Coda et al., 2017; Chan et al., 2019). Moreover, dry fractionation-derived faba bean protein concentrate has been demonstrated to be suitable for meat-like products (do Carmo et al., 2021). Fibrous textured meat analogues were successfully produced using high moisture extrusion of commercial faba bean concentrate (56% protein) or isolates prepared by aqueous fractionation (88% protein) (Ferawati et al., 2019).

The increased number of studies on incorporating faba bean protein fractions into various food products suggests that this ancient pulse is nowadays consumed in new and alternative ways. To promote the use of faba bean as reliable food ingredient, it is crucial to produce high-quality seed-derived ingredients. Plant breeding can significantly contribute to developing optimal faba bean varieties tailored for modern food applications.

### 1.2 Faba bean ideotypes for modern food applications are lacking in the variety portfolio

In the period 1960-1990, Europe witnessed a severe decline in the faba bean cultivation area. The primary reasons include small economic margins for farmers, economic competition with soybean, as well as low and unstable yields caused by biotic and abiotic stress (Flores et al., 2013; Karkanis et al., 2018; Mínguez and Rubiales, 2021). The lack of interest in European faba bean cultivation in the period 1960-1990 resulted in a relatively brief breeding history with limited investments, starting only in the 1980s in Europe. Seed production has been traditionally relegated to small farms, where landraces have been selected based on their agronomic performance at a local scale (Duc et al., 2010), representing the main genetic material cultivated. Consequently, there is a restricted number of varieties registered in the European catalogue in comparison to field pea and soybean (European Commission, 2023). The main varieties available in the market belong to the botanical groups *Vicia faba* var. *major* Harz. (broad bean) and *Vicia faba* var. *minor* Beck. (field bean). Broad bean and field bean mainly exhibit differences in the morphology of the seeds (i.e., seed size), with broad bean possessing larger seeds than field bean (Muratova, 1931). In Europe, broad beans are primarily utilised for food purposes, while field beans serve as animal feed or green manure (Pasqualone et al., 2020).

Following 1990, faba bean yield started to increase because of genetic selection. Breeding has been conducted to increase tolerance to winter climate conditions (frost hardiness), vernalization requirement and photoperiodic induction (Arbaoui et al., 2008). As a result, spring and winter types are available. Winter-type varieties are typically sown in autumn in cold-temperate regions, including the Mediterranean basin or some mild climates of Australia and China (Flores et al., 2012; Mínguez and Rubiales, 2021). Farmers are particularly attracted by the possibility of cultivating the winter faba bean in a wider area in Europe, even in colder climates (Neugschwandtner et al., 2019). Certain cultivars with particularly enhanced hardening responses are currently cultivated in the UK and other high-latitude regions. Spring-type varieties, on the other hand, do not require vernalization or hardening response, thus are currently the most preferred in cold climates (Flores et al., 2012; Mínguez and Rubiales, 2021).

Improving seed quality has not been a major target for breeding, mostly because there was no impulse from the food industry. The breeding efforts in seed quality have been mostly directed to contents of tannins and the alkaloids vicine and convicine (V-C) (Gutierrez and Torres, 2019; Bjornsdotter et al., 2021; Tacke et al., 2022). At the moment, the European catalogue of registered varieties includes four types with improved quality based on the reduced tannin content in the seed testa (outer protective layer of the bean) and the lower levels of V-C in the cotyledons (the part of the seed embryo that will become the first leaves of the plant upon germination). These four types refer to high or low levels of tannins, and high or low levels of V-C. The word “fevita” usually refers to the simultaneous combination of low tannins and V-C, after the first variety of this type was registered in the French catalogue under the name of FEVITA^®^ in 2004 (Crépon et al., 2010; Zong et al., 2019). Despite the release of varieties with low antinutritional compounds on the market, varieties with high levels of tannins and V-C are still the most cultivated.

Faba bean is gaining popularity in several countries as an ingredient for plant-based products. However, faba bean ideotypes for modern food applications (i.e., plant-based meat analogues) are lacking in the market. The development of improved varieties with good techno-functional properties (i.e. solubility, emulsifying, foaming ability, etc.), together with nutritional improvement, is pivotal to expanding the use of faba bean ingredients in the food industry. Interestingly, new varieties could be bred for protein components and amino acid composition, which are intrinsic components impacting the techno-functionality of the products during processing (Rahate et al., 2021; Augustin and Cole, 2022).

## 2 Defining reduced off-flavor as a quality trait in faba bean breeding

One of the major obstacles to adopting a diet with reduced meat consumption is the lack of familiarity with meat-alternative products and their limited aroma, taste and texture attractiveness (Wang et al., 2022).

Flavour is a multisensory perception defined by taste and aroma, which are influenced by the food matrix and its complex physico-chemical affinity with flavor-active chemical compounds (Tournier et al., 2007; Roland et al., 2017). Aroma refers to the perception of volatile odour-active compounds via receptors in the nose (Czerny et al., 2008). The main aroma features of pulses are beany, grassy, and earthy (Wang et al., 2021). Taste is perceived via receptors on the tongue and in the oral cavity and results from non-volatile compounds present in the grains. The taste of pulses is often reported as bitter and astringent (Roland et al., 2017; Sharan et al., 2021). A specific flavor can be perceived as pleasant or unpleasant (“off-flavor”) by consumers, depending on the intended final product. For example, the typical faba-bean-like flavor is unpleasant in meat or dairy alternative analogues, as consumers expect tastes like meat, milk, and cheese rather than beans. Consequently, off-flavors hinder consumer preference and acceptance of faba bean in modern plant-based products (Wang et al., 2022). Ideal faba bean ingredients should be as bland as possible in terms of flavor.

Prior to starting breeding programs and setting breeding objectives, plant breeders need understanding about off-flavor molecules and which of them highly impact on off-flavor in the final ingredients. In this way, marker compounds for screening for unpleasant flavors can be defined. It is important to understand that not all compounds detected in a food or food ingredient contribute to its flavor, and determining the real impact of the diverse aroma and taste compounds on perception typically involves laborious methods. Furthermore, the sensory impact of compounds is not solely based on their concentration, but it also relies on their specific sensory thresholds. Some compounds are detectable by some consumers at ultra-trace levels (Ridgway, 2015). The importance of volatile compounds on flavor is usually ranked by calculating the so-called odour activity values (OAVs), which simultaneously account for their concentration and sensory threshold. Volatile compounds with high OAVs are often essential to the aroma, unless they are suppressed in the respective matrix (Grosch, 2001). However, it is worth mentioning that the sensory thresholds of compounds vary across different mediums or the matrix in which these compounds are incorporated. For example, the taste threshold of kaempferol is 20 mg/kg in 5% aqueous ethanol, but it is almost 50 mg/kg in beer (Van Gemert, 2003). Another approach to rank the importance of flavor compounds in a food matrix involves taste dilution or aroma dilution experiments. In aroma dilution experiments, gas chromatography-olfactometry (GC-O), which combines the chromatographic separation of compounds with human sensory detection, is used. The result of this analysis is expressed as a ‘flavor dilution factor’ (FD-factor) (Grosch, 2001). In taste dilution experiments, a sensory panel is involved, and the result is expressed as ‘taste dilution factor’ (TD factor) (Frank et al., 2001). Higher dilution factors imply a greater impact of the respective compound on the flavor of the food.

It is worth noting that the identification of key off-flavor compounds depends on several factors. For instance, different varieties of faba exhibit a different level of off-flavors compounds (Akkad et al., 2021), but the difference in off-flavor can also be caused by the specific processing applied to the seeds (Tuccillo et al., 2022). Currently, faba bean seeds are processed to produce flour or protein concentrates and isolates. Protein concentrate and protein isolate are two types of protein-enriched fractions (Augustin and Cole, 2022). Protein concentrates typically contain about 60-80% protein, while protein isolate, due to additional processing to remove nearly all non-protein components, results in a purer protein content of around 90-95% (Ma et al., 2022). Tuccillo et al. (2022) recently demonstrated that flour, protein concentrate and protein isolate can have different off-flavors and off-flavor intensities. This is likely linked to the different protein content of different ingredients, as protein play as a carrier of flavor compounds, but is also influenced by the distinct handling processes of the seeds (e.g., removal off the seed coats, temperatures etc.) and by the oxidative reactions occurring in the meantime.

The dominant flavor of faba bean has been traditionally described as dried pea flavor, bitter aftertaste and unpleasantly fruity (Seidel, 1976; Schultz et al., 1988). Specific research on the off-flavor properties of faba bean is scarce compared to soybean and pea, partially because the use of faba bean as a plant-based food ingredient is more recent. For the investigated pea matrices, key off-flavor compounds have been pointed out. Taste dilution experiments have been conducted for bitter compounds in pea protein isolates (Glaser et al., 2020). Moreover, the use of GC-O and OAV calculations using literature thresholds in water have been reported for pea milk (Zhang et al., 2020). For faba bean, Akkad et al. (2021) conducted chemical analyses of volatile compounds in flour and determined key volatile flavor compounds by calculating relative OAVs using literature thresholds in air. In another study, Tuccillo et al. (2022) combined sensory analysis with chemical analyses of volatile and non-volatile compounds, employing statistical methods to identify compounds in faba bean ingredients and extrudates that are correlated with off-flavor. The most recent study, by Karolkowski et al. (2023a), performed gas chromatography mass spectrometry (GC-MS) and GC-O following solvent assisted flavor extraction (SAFE) of various faba bean fractions, including flour, starch, and protein. However, to the best of our knowledge, no flavor dilution or taste dilution experiments have been reported yet for faba beans, and knowledge gaps regarding key off-flavor compounds (mainly non-volatile) are still relatively large. Therefore, educated guesses need to be taken to determine which compounds potentially have an impact on faba bean off-flavor, based on their presence and abundance, their sensory characteristics, and their impact known for crops like pea and soy.

In the following subparagraphs, we discuss a summary of molecules potentially involved in off-flavor development in faba bean and their origins. **Table 1** presents a shortlist of molecules that we consider suitable as marker compounds for screening faba bean ingredients for potential off-flavors. Our observations are supported by literature, such as key compounds determined by relative OAV (Akkad et al., 2021) and by GC-O (Karolkowski et al., 2023b) in faba bean, supplemented by observations in our own laboratories. A marker compound is not necessarily the one with the most significant impact on the flavor. It can also be a compound produced in large amounts and capable of indicating the occurrence of off-flavors-related chemical reactions. For this reason, the amounts of oxidation compounds formed when C18:1, C18:2, and C18:3 are oxidized (Belitz et al., 2009) were also included in the final choice of the makers for volatile compounds. It is important that the marker compounds for off-flavors are easy to detect, acting as proxy of other chemicals that might have a more substantial impact on flavor but are less detectable or whose detectability is affected by extraction techniques.

**Table 1:** Shortlist of marker compounds for potential off-flavor in faba bean ingredients.

Flavor type	Pathway	Compound ^a^	Flavor characteristics	Perception threshold
Aroma (volatile compounds)	Polyunsaturated fatty acid (PUFA) oxidation	hexanal	apple, cut grass, fresh, fruit, grass, green, oil	0.043 mg/L ^b^
		nonanal	citrus, cucumber, fat, floral, green, metal, paint, pungent, rubber, soap, sweet	0.022 mg/L ^b^
		(E)-2-heptenal	almond, fat, fruit, metal, soap	0.018 mg/L ^b^
		(E)-2-nonenal	beany, cucumber, cut grass, fat, green, hay, oil, paper, sour, stale, tallow, watermelon	0.0001 mg/L ^b^
		(E,Z)-2,4-heptadienal	fat, fried, nut	0.0035 mg/L ^b^
		(E,E)-2,4-nonadienal	deep fried, fat, geranium, green, hay, metal, nut, seaweed	0.00006 mg/L ^b^
		(E,Z)-2,6-nonadienal	cucumber, green, lettuce, melon, wax	0.000036 mg/L ^b^
		1-octen-3-ol	earth, fat, floral, green, herb, mould, mushroom, yeast	0.097 mg/L ^b^
		2-pentylfuran	butter, floral, fruit, green, green bean, metal, rubber, sweet	0.0048 mg/L ^b^
	Amino acid degradation	3-methylbutanal	acrid, almond, chocolate, cocoa, corn flakes, fermented, malt, pungent, sweat, sweet	0.06505 mg/L ^b^
		Benzaldehyde ^e^	almond, berry, bitter, bitter almond, burnt sugar, cherry, fruit, malt, roasted pepper, spice, sweet	0.58 mg/L ^b^
		2-methoxy-3-isopropyl-(5 or 6)-methylpyrazine	Earthy, spicy, plastic, hay ^c^	0.00005 mg/L^d^
	Others	linalool	bergamot, coriander, floral, flower, grape, lavender, lemon, rose	0.0051 mg/L ^b^
		dimethyldisulphide	cabbage, garlic, meat, onion, putrid, sour, sulfur	0.051 mg/L ^b^
Taste and mouthfeel (non-volatile compounds)	Saponin biosynthesis	saponin βg	Bitter	<2 mg/L ^f^
		saponin Bb	Bitter	8 mg/L ^f^
	Tannin biosynthesis	epicatechin	Astringent Bitter	270 mg/L ^g^ 270 mg/L ^g^
		procyanidin B2	Astringent Bitter	110 mg/L ^g^ 280 mg/L ^g^
		procyanidin C1	Astringent Bitter	260 mg/L ^g^ 347 mg/L ^g^
	Hydroxycinnamic acids biosynthesis	p-coumaric acid	Astringent	23 mg/L ^g^
		ferulic acid	Astringent	13 mg/L ^g^
	Flavonols biosynthesis	quercetin	Bitter, astringent	10 mg/L ^h^
		kaempferol	Bitter, astringent	20 mg/L ^h^
	Polyunsaturated fatty acid (PUFA) oxidation	11,12,13-trihydroxyoctadec- 9-enoic acid	bitter	42.96 mgl/L ^i^
		1-linoleoyl glycerol	bitter	24.81 mg/L ^i^
		linoleic acid	bitter	260.81 mg/L ^i^
		α-linolenic acid	bitter	77.9 mg/L ^i^

a the provided list of chemical compounds is a representative selection of many more molecules that are usually produced.

b The reported perception thresholds are the average values of the flavor in water thresholds reported in the VCF online database.

c The flavor characteristics refers to Zhang et al., 2020 (due to lack of sensory description in the VCF online database).

d The reported threshold is odour in water threshold (due to the lack of provided flavor in water thresholds).

e Benzaldehyde can be produced by the degradation of both free fatty acids and amino acids (Akkad et al., 2019); Oomah et al., 2014).

f The reported threshold refers to Heng et al. (2006) and it is in water.

g The reported threshold refers to Hufnagel and Hofmann (2008a) and it is in water.

h The reported threshold refers to Dadic and Belleau (1973) and it is in 5% ethanol.

i The reported threshold refers to Glaser et al. (2020) and it is in water. The original values were in mmol/L and here are converted in mg/L.

The table shows the flavor type (aroma or bitter taste/astringent mouthfeel), the originating pathway for each compound, chemical breeding traits (referred as compound), typical perception attributes (referred as flavor characteristics), and the perception threshold, which indicates the minimum concentration required for the compound’s detection.

### 2.1 Unpleasant aroma is associated with lipids oxidation, amino acids degradation, and other chemical pathways

The unpleasant aroma of faba bean protein fractions is associated with the release of certain volatile compounds originating from non-volatile precursors like lipids, amino acids, and carbohydrates. The chemical reactions leading to the development of aroma compounds mainly occur during harvesting, postharvest processing, and storage (Roland et al., 2017). Even though faba bean is not an oil crop and has a low-fat content (0.5-2% dry matter), it is still prone to developing off-flavors as a result of lipid oxidation (Wang et al., 2014). In fact, some lipid-derived compounds can be detected by the human nose at extremely low concentrations (Akkad et al., 2021). Polyunsaturated fatty acids (PUFA) like oleic acid (C18:1), linoleic (C18:2) and linolenic (C18:3) are readily oxidised to hydroperoxides, which are subsequently degraded in volatile secondary oxidation products (i.e., aldehydes, ketones, alcohols, aromatic hydrocarbons, furans, etc.), some of which being exceptionally odorous compounds (Decker et al., 2012). The formation of beany flavor is primarily attributed to aldehydes and dienals produced through PUFA degradation catalysed by LOX. Akkad et al. (2021) reported (in order of decreasing Relative OAVs) nonanal, octanal, hexanal, 3-methylbutyric acid, decanal, 3-methylbutanal, 1-octen-3-ol, and others as key volatile compounds in faba bean flours of 4 faba bean cultivars, based on the calculation of relative odour activity values. The type of volatile compounds produced from the oxidation of PUFA mainly depend on the nature of the fatty acids involved in the reaction. The oxidation of oleic acid oxidation leads to the formation of 8-, 9-, 10-, and 11-hydroperoxides, and subsequentially to many secondary compounds such as pentanal, octanal, hexanal, and heptanal, among others (Shahidi and Hossain, 2022). The oxidation of linoleic acid results in two distinct stereoisomers of both 9- and 13-hydroperoxides. As an example, the volatile 2,4-decadienal primarily originates from 9-hydroperoxide, whereas hexanal is primarily produced from 13-hydroperoxides. Linolenic acid, on the other hand, oxidises to generate 9-, 12-, 13-, and 16-hydroperoxides (Karolkowski et al., 2021). Linoleic and linolenic-derived compounds have more impact on off-flavor compared to those derived from oleic acid.

Lipid peroxidation occurs via autoxidation or enzymatic oxidation which requires the presence of lipoxygenase. The susceptibility of fatty acids to lipid peroxidation augments proportionally with the degree of unsaturation (Belitz et al., 2009). Oleic acid results more stable than linoleic and linolenic acid. Linoleic acid is about ten times more susceptible to autooxidation and linolenic acid twenty times more (Decker et al., 2012). Moreover, lipoxygenase selectively oxidizes linoleic and linolenic acids, while oleic acid remains unaffected by this enzymatic process.

Lipid oxidation is not the only source of unpleasant aroma development in faba bean. Akkad et al. (2019) suggested that the high protein content of faba bean varieties may impact their aroma profile. Amino acid degradation can generate a myriad of volatile compounds, such as branched-chain compounds, benzene aldehydes, alcohols, acids, esters, nitrogen, and sulfur compounds (Karolkowski et al., 2021). Amino acid-derived volatiles, including 3-methylbutanal and 3-methylbutanoic acid (leucine-derived), 2-methylbutanal (isoleucine-derived), and benzacetaldehyde (phenylalanine-derived) were detected in faba bean flour (Akkad et al., 2019). In addition to the degradation pathway, amino acids generate odor-active compounds through reactions with sugars and carbonyls induced by the effect of temperature (Maillard reaction) (MacLeod and Ames, 1988).

Other compounds, such as terpenes, can also influence the aroma profile of faba bean. For example, Akkad et al. (2019) detected limonene, which typically has a citrus-like odour at high concentrations. Additionally, the presence of linalool, alpha-pinene, delta-3-carene was also reported (Akkad et al., 2019; Tuccillo et al., 2022). Naturally occurring methoxypyrazines were hypothesised to potentially play a role in off-flavor development in V. faba due to their key role in the aroma of green peas and their extremely low odour thresholds (Sharan et al., 2021). However, due to their extremely low concentrations at which they are odour-active, they are rather detected by the nose, than by GC-MS. Therefore, while GC-MS measurements of faba bean fractions failed to detect any methoxypyrazines, the GC-O measurements-which involve smelling the separated volatiles-revealed the presence of 3-isopropyl-2-methoxypyrazine and 3-isobutyl-2-methoxypyrazine (Karolkowski et al., 2023b).

### 2.2 Unpleasant taste is associated with polyphenols, saponins and lipid-derived compounds

The bitter and astringent unpleasant taste of legumes is associated with non-volatile compounds present in seeds. The information on the role of non-volatile compounds in the taste of faba bean is scarce, but it is widely accepted that polyphenols (such as phenolic acids, flavones, flavanones, flavonols, flavan-3-ols, and proanthocyanidins), saponins, amino acids and peptides are bitter and/or astringent compounds (Drewnowski and Gomez-Carneros, 2000; de Camargo and Schwember, 2019; Iwaniak et al., 2019), and also many non-volatile lipid oxidation compounds and oxidised phospholipids taste bitter (Sessa and Rackis, 1977; Glaser et al., 2020).

Phenolic compounds are usually either measured as total phenolic compounds with the use of the Folin-Ciocalteu assay, reporting gallic acid equivalents (GAE) measured in a spectrometric way (e.g., in Tuccillo et al., 2022), or they are measured individually by chromatographic methods (e.g., in Abu-Reidah et al., 2017). Various phenolic compounds from the classes of phenolic acids, flavanols, procyanidins, prodelphinidins, flavanones, flavonols, flavones, and others, have been detected in *Vicia faba* (Turco et al., 2016; Abu-Reidah et al., 2017). Many of these flavonoids activate bitter taste receptors (Roland et al., 2013), so it can be expected that they impact off-taste in this crop. For example, the flavonols quercetin and kaempferol are present in faba bean (Troszyńska et al., 2006) and are bitter (Drewnowski and Gomez-Carneros, 2000; Roland et al., 2013). The phenolic acids vanillic, caffeic, p-coumaric, and ferulic acid have been perceived as astringent (Hufnagel and Hofmann, 2008a) and have been detected in faba bean (Troszyńska et al., 2006). However, which of these phenolic compounds impacts the perception of bitterness and astringency in faba bean depends on their concentrations and the presence of other compounds in the matrix (Roland et al., 2017). In fact, free phenolics have been recently associated to the strong taste, bitterness, and mouth-drying sensation of faba bean protein contrate (Tuccillo et al., 2022), but the understanding of which specific individual compounds links to this off-taste is lacking.

An important class of polyphenols in faba bean is represented by the tannins. Tannins are mainly classified into two categories based on their reactions with hydrolytic agents: condensed tannins and hydrolysable tannins. Hydrolysable tannins have a central carbohydrate core esterified to phenolic carboxylic acids such as gallic acid and ellagic acid and are usually referred to as ellagitannins and gallotannins. Condensed tannins are mainly polymerised products of flavan-3-ols. The most occurring classes are procyanidins, formed by (epi)catechins, and prodelphinidins, formed by (epi)gallocatechins (Appeldoorn, 2009). Tannins are often determined as total tannins, using e.g. a vanillin assay and determining the Catechin Activity Equivalents (CAE) in a spectrometric way. Despite naming “tannins” in many articles, the literature about which tannins exactly are present in faba beans is scarce and few articles were published. Merghem et al. (2004) identified different types of procyanidins in faba bean, namely 2 types of procyanidin A-dimers (without being able to specify which exact A-dimers), the procyanidin dimers B1, B2, and B3, the trimer C1. A subsequent study (Abu-Reidah et al., 2014), performed metabolic profiling of faba bean seeds and tentatively identified the procyanidin dimers B1, B2, B3, and B4, and diverse prodelphinidin dimers. Tannins are bitter and/or astringent depending on their degree of polymerization. Lower-weight tannins generally have a more pronounced bitter taste, while those with a higher degree of polymerization tend to be more astringent (Peleg et al., 1999; Soares et al., 2020). According to Peleg et al., 1999, flavanol monomers (for example catechin and epicatechin) are more bitter than astringent, and the dimers and trimers (for example procyanidins) are more astringent than bitter (Peleg et al., 1999). However, a partially contradictory effects were observed by Hufnagel and Hofmann (2008a, b). In taste receptor assays, lower bitterness of the monomer epicatechin compared to the procyanidin trimer C2 was shown (Soares et al., 2013). Tannins have historically attracted the attention of faba bean breeders, as they are considered major antinutritional factors (Guevara Oquendo et al., 2022). However, condensed tannins have been shown to evoke positive effects on human cardiovascular health as well (Appeldoorn, 2009).

Saponins are known to contribute to the bitter, astringent, and metallic taste of pea and soybean (Price and Roger Fenwick, 1984; Heng et al., 2006). They are natural compounds found in many plants, especially legumes. Saponins are triterpenoid glycosides, in which an apolar sapogenol backbone is substituted with one sugar chain (at sapogenol B and sapogenol E) or two sugar chains (at sapogenol A) (Decroos et al., 2005). The length and composition of the sugar chain determines the exact type of saponin. B-type saponins are the most common in legumes. A-type saponins are reported in soy (Sundaramoorthy et al., 2019a), but not in pea and faba bean. In the intact plant tissue, B-type saponins occur as conjugates of DDMP (2,3-dihydro-2,5-dihydroxy-6-methyl-4H-pyran-4-one). They are easily degraded to non-DDMP conjugated saponins upon processing. Soyasaponin αg, βa, βg, γa, and γg- belong to DDMP-saponins, while soyasaponins Bb (in another nomenclature called saponin I), Bc (II), Bb’(III), Bc’(IV), Ba (V) etc. belong to non-DDMP conjugated saponins (Barakat et al., 2015) (Singh et al., 2017). It has been shown in sensory experiments with saponins extracted from pea that these saponins are bitter and that DDMP-saponin βg is more bitter than saponin Bb (Heng et al., 2006), but no information related to the impact of saponins on the bitter taste of faba bean is present in literature.

Alkaloid content affects bitterness in food products such as lupins, which are classified into “bitter” or “sweet” varieties based on this attribute. Faba bean contains two pyrimidine glucoside alkaloids, vicine and convicine (V-C), yet their flavor characteristics have remained unexplored (Karolkowski et al., 2023a). V-C compounds may have been overlooked in recent flavor studies due to their absence in other major legumes, especially soybean. Moreover, their potential toxicity to some consumers might hamper sensory tests. When compounds are toxic and sensory tests are unfeasible, there is an alternative to test for bitterness, namely by *in vitro* taste receptor assays. However, although many toxic compounds (e.g., the alkaloid strychnine) have been tested on receptor level (Meyerhof et al., 2010), vicine or convicine have not been reported to be tested yet. Tuccillo et al. (2022) reported a correlation between V-C presence, bitterness, and mouth dryness, but this correlation also accounted for free phenolics and specific amino acids (Tuccillo et al., 2022). Given that V-C are expected to be bitter, the ongoing genetic efforts aiming to entirely remove their content for nutritional reasons (Bjornsdotter et al., 2021) will also be beneficial to the taste of the seeds.

Off-taste cannot only be evoked by compounds inherently present in the seeds like described above, but it can also be evoked by non-volatile lipid oxidation compounds. In pea protein isolates, it has been shown that bitterness was mainly caused by four trihydroxy-octadecenoic acid isomers (e.g. 9,10,11-trihydroxy-octadec-12-enoic), four hydroxy-octadecadienoic acid isomers (e.g. (10E,12E)-9-hydroxyoctadeca-10,12-dienoic acid), one oxo-octadecadienoic acid, one octacosatetraen, 1-linoleoyl glycerol, 2-hydroxyoleic acid, 2-hydroxypalmitic acid, α-linolenic acid, and linoleic acid (Glaser et al., 2020). Some of these compounds showed very low sensory threshold values (e.g., 0.08 mmol/L for 9,10,13-trihydroxyoctadec-12-enoic acid). The impact of non-volatile lipid oxidation compounds on bitterness has been confirmed in other crops. In wheat, two trihydroxy octadecenoic acids (pinellic acid, 8R*,9R*,10S*-trihydroxy-octadec-6Z-enoic acid), one trihydroxy-octadecadienoic acid (9S,12S,13S-trihydroxy-octadeca-10E,15Z-dienoic acid) and 1-(octadeca-9Z,12Z-dienoyl)-sn-glycero-3-phosphocholine have been identified as bitter, and associated with a dislike for the whole wheat breads (Cong et al., 2021). Analysis of wheat mutant lines, with the lipoxygenase gene disrupted, showed a significant reduction (88–93%) in bitter compound production when compared to the unaltered, or wild type, control. This establishes that the activity of the lipoxygenase enzyme plays a crucial role in creating the bitter taste from trihydroxy fatty acids in wheat (Cong et al., 2021). For faba bean or its ingredients, such bitter non-volatile lipid oxidation compounds have not been reported in literature yet. Given the presence of PUFAs and lipoxygenases in this crop, it can be expected that these compounds can be formed in faba bean as well. However, it remains to be established for faba bean ingredients which of all the potentially bitter and astringent compounds really contribute to the bitter and astringent sensation.

## 3 Plant breeding as a strategy to establish *Vicia faba* as plant-based food ingredient

Various thermal, chemical, or enzymatic strategies have been investigated to bridge the flavor gap in plant-based proteins by eliminating, modifying, or masking the development of undesirable taste and aroma (Tangyu et al., 2019; Xu et al., 2020; Mittermeier-Klessinger et al., 2021; Sontag-Strohm et al., 2021). However, post-harvest processing is often costly and energy-intensive, and sometimes the off-flavor is persistent despite the measures taken. An alternative approach is the breeding of faba bean varieties that exhibit low levels of off-flavor precursors, off-flavors, or enzymes that contribute to off-flavor development (Roland et al., 2017). Despite the identification of molecules having an impact on the aroma and possibly on the taste and astringent mouthfeel of faba bean ingredients in previous studies (Oomah et al., 2014; Akkad et al., 2019; Karolkowski et al., 2023a), a knowledge gap persists regarding specific marker compounds that could serve as proxies for off-flavor development in breeding programs. Determining and characterising these compounds further, along with the pathways responsible for their production, is essential for devising effective plant breeding strategies aimed at preventing or minimising off-flavors in faba bean.

It is important to specify that preventing the formation of off-flavors is a breeding target for the use of faba bean as ingredient in plant-based dairy or meat analogues, but not for other applications where seeds are cooked or consumed freshly. Breeding efforts to improve seed quality in faba bean must consider the significant variation in consumer preferences influenced by cultural and personal factors (Szenderak et al., 2022). Some consumers might still prefer the traditional usage of the seeds, where the distinctive and unique flavor is actually a positive and desired feature. Breeding for quality in this context focuses on enhancing the nutritional properties of the seeds rather than removing the typical taste and aroma. Important breeding targets include the removal of antinutritional compounds such as tannins, vicine and convicine, phytic acid, lectins, trypsin inhibitors, and oligosaccharides (e.g., raffinose, stachyose), and enhance the protein content. Additionally, cooking time is increasingly recognised as an essential trait since traditional home cooking of faba bean involves extended time to achieve satisfactory softness and palatability (Dhull et al., 2022). Therefore, faba bean breeding should aim to develop varieties that cater to different product end-uses, including those for sustainable and nutritious alternatives to animal protein sources.

The following paragraphs illustrate the candidate pathways that are critical to major off-flavors development and how they can be targeted with breeding aimed at improving the quality of faba bean for plant-based meat and dairy analogues.

### 3.1 Targeting lipid-oxidation pathways to reduce beany flavor and lipid-derived bitterness

#### 3.1.1 Disrupting *LOX* genes leads to reduced oxidation rate of fatty acids

The *LOX* genes are known to regulate the enzymatic oxidation rate of polyunsaturated fatty acids, such as linoleic acid (C18:2) and linolenic acid (C18:3), through the expression of the lipoxygenase enzyme (Shahidi and Oh, 2020). Consequently, disrupting *LOX* genes would likely result in a decrease in the oxidation rate, reducing the amount of derived volatile and non-volatile compounds causing unpleasant aroma and taste, respectively.

**Figure 2:**
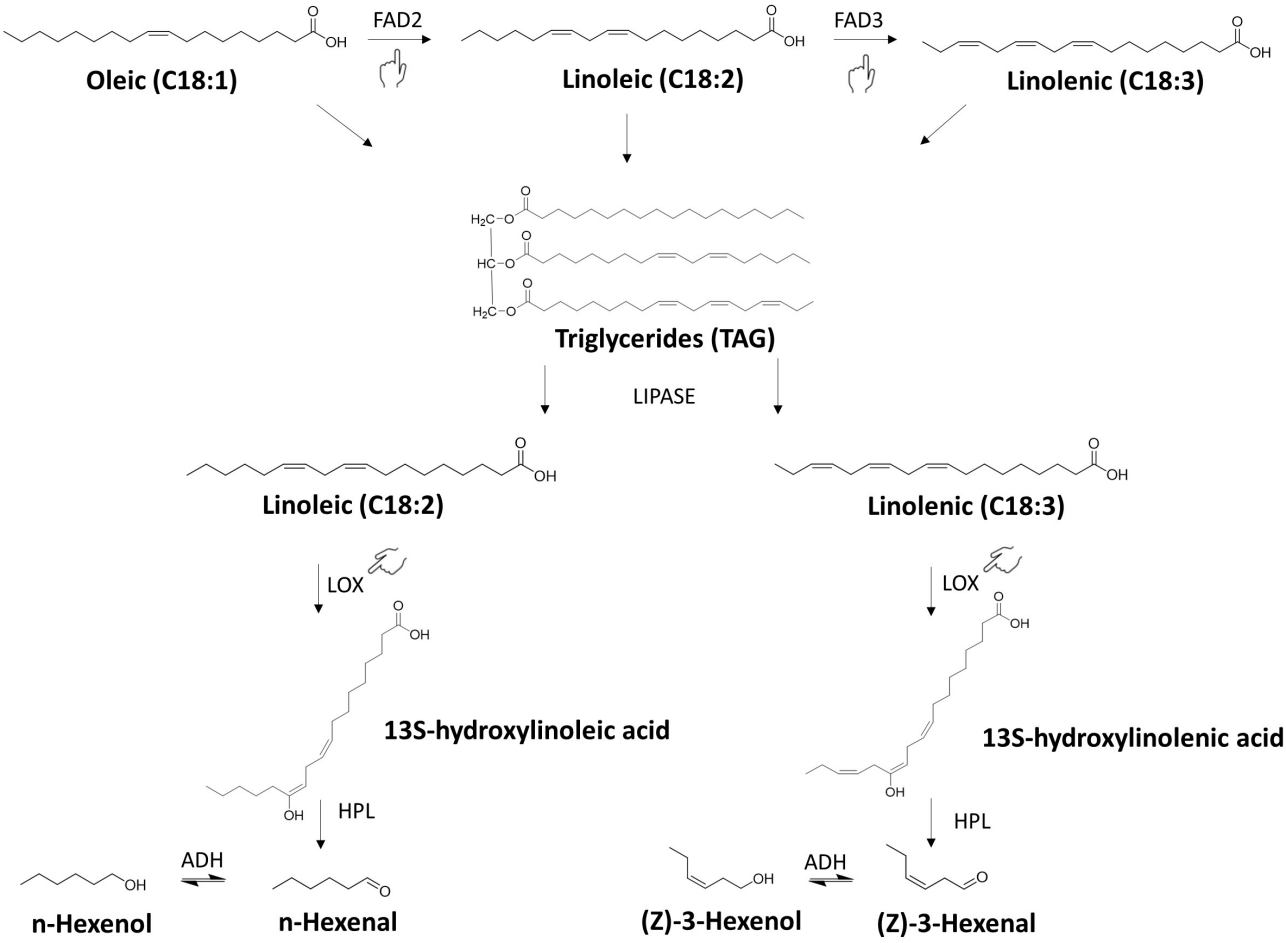
Biosynthesis of lipid-derived off-flavors. The figure depicts a simplified representation of the biosynthesis of lipid-derived off-flavors. The process begins with oleic acid (C18:1) serving as precursor for the synthesis of linoleic acid (C18:2) and linolenic acid (C18:3), mediated by enzymes FAD2 and FAD3 respectively. A portion of the produced polyunsaturated fatty acids is assembled into triacylglycerides (TAGs). Free polyunsaturated fatty acids, released by lipase action on TAGs, undergo oxidation by lipoxygenase enzymes (LOX) to form hydroperoxides. Hydroperoxides are decomposed into various compounds, including aldehydes, alcohols, ketones, and hydrocarbons, through the activity of hydroperoxide lyase (HPL) and alcohol dehydrogenase (ADH). In the figure, as example, n-hexanal and (Z)-3-hexenal represent the class of aldehydes, while n-hexanol and (Z)-3-hexenol represent the class of alcohols. However, the real molecules formed, and the number of reactions happing are numerous. The figure highlights (pointing hand) *FAD2*, *FAD3*, and *LOX* as target genes for breeding efforts aimed at reducing the fatty acid oxidation rate. The acronym of each key enzyme is reported along the pathway: FAD2 and FAD3, fatty acid desaturase 2 and 3; LOX, lipoxygenase; HPL, hydroperoxide lyase; ADH, alcohol dehydrogenase.

Lipoxygenases selectively act on these fatty acids containing a 1-cis,4-cis-pentadiene unit producing the odourless stereospecific hydroperoxides. The latter are highly unstable and are in turn decomposed into a variety of compounds, including carbonyl compounds, alcohols, ketones, furans, epoxy compounds and hydrocarbons, among others, through the action of hydroperoxide lyase (HPL) and alcohol dehydrogenase (ADH) (see **Figure 2**) (Baysal and Demirdoven, 2007; El Hadi et al., 2013). The stereo-specificity of LOX enzymes has significant implications on the diversity of short-chain aldehydes, alcohols, and hydrocarbons produced, and thus on the final aroma (Karolkowski et al., 2023a). Lipoxygenases found in plants predominantly demonstrate regiospecificity towards the 9 or 13 carbon positions (Belitz et al., 2009). Additionally, LOX-catalysed reactions (but also auto-oxidation) contribute to the formation of lipid-derived non-volatile compounds. The hydroperoxides that are produced during the oxidation process can be transformed into various epoxy and hydroxy fatty acids by the action of peroxygenase (POX). This represents an alternative pathway to their conversion into volatiles by hydroperoxide lyase (HPL) and alcohol dehydrogenase (ADH) (Feussner and Wasternack, 2002). Epoxy and hydroxy fatty acids were identified in pea and oat and shown to be bitter in pea (Yang et al., 2019; Glaser et al., 2020; Glaser et al., 2021).

*LOX* family genes are conserved among legumes. In fact, lipid-oxidations products, (i.e, hexanal, 1-hexanol and 2-pentylfuran, etc.) are considered markers for “beany” flavor in other legumes such us chickpea, lentil, and yellow pea (Xu et al., 2019). Additionally, aldehydes derived from lipids are well known to cause the green, earthy, fatty, leafy aroma of peas and soybean (Seo and Hummel, 2012; Akkad et al., 2019). Two lipoxygenase isoenzymes (BBL-1/BBL-2) have been isolated in faba bean, but no studies to assess whether the presence of null or mutant alleles has a downstream effect on the flavor profile have been reported (Clemente et al., 2000).

The expression of these enzymes could vary among different cultivars, potentially accounting for the differences in odour-active compounds across them. In pea, for instance, a variation in lipoxygenase activity (as much as 70%) among three distinct commercially available pea types has been reported (Haydar and Hadziyev, 1973; Trindler et al., 2021). In addition, it has been shown that LOX differential activity depends on genetic variation in soybean (Mandal et al., 2014). If such genetic variations exist in faba bean, it could potentially serve as a basis for breeding initiatives aimed at creating new cultivars with reduced beany flavor.

Dysfunctional LOX pathways lead to reduced oxidation rate and are of interest for breeding applications in pulses. Lately, the newly developed soy genotype NRC132, which features *lipoxygenase2* inactivation, has shown that *LOX*-free variants can exhibit diminished off-flavor in soy-milk production (Kumar et al., 2021). This development presents new possibilities for incorporating soybean into a broader range of food items. Several *lipoxygenase*-free soybean varieties are already available for cultivation in the USA (i.e., IA1008LF, IA2053LF, IA2076LF, IA2104LF, IA3027LF, IA3045LF, IA3051LF, etc.) (Yu et al., 2016). Based on these findings, the development of lipoxygenase-free faba bean seeds is envisioned in the near future.

Although some LOX enzymes do not require the prior release of fatty acids from their storage forms, the hydrolysis of acyl lipids to free fatty acids (FFAs) is generally required for their oxidising activity in plants (Lampi et al., 2020). Lampi et al. (2020) have emphasised the need to control endogenous lipase in faba bean during food processing due to its high lipid hydrolysis activity, which can also impact exogenous lipids in food products. The investigation of lipase alleles’ functionality is important for understanding off-flavor development in view of genetic improvement of this trait. However, lipases are essential for the utilization of storage triglycerides by developing seeds (Kanai et al., 2019), thus their genetic removal may be more critical than other candidate genes hereby proposed.

#### 3.1.2 Disrupting *FAD* genes causes lower unsaturation of fatty acids and increased oil stability

Ideally, faba bean cultivars to produce protein fractions for meat and dairy analogues ought to exhibit decreased levels of C18:2 and C18:3, thereby diminishing the available substrate for lipoxygenase activity. Oil stability, characterised by resistance to oxidation, is affected by the ratio between C18:1, C18:2, C18:3 in the total oil composition. Enhancing the level of C18:1 while reducing C18:2 and C18:3 is an important breeding target to reduce fatty acid oxidation.

In several species, *Fatty Acid Desaturase* (*FAD*) genes are responsible for adding double bonds to the oleic acid (C18:1) to form linoleic (C18:2) and linolenic acids (C18:3) (Zhang et al., 2008). In particular, FAD2 (omega-6 fatty acid desaturase) is the key enzyme that catalyses the desaturation of C18:1 into C18:2 in roots and developing seeds of oil crops (Traore and He, 2021), while FAD3 (omega-3 fatty acid desaturase) subsequentially converts C18:2 into C18:3 (**Figure 2**) (Dar et al., 2017). The faba bean research community has previously not focused on investigating these genes, as they are typically not considered pertinent in non-oil crop research. However, breeding efforts should be conducted to develop high-oleic faba bean varieties less prone to oxidation. Evidence from soybean research indicates that combining two mutant isoforms of *fad2* (*GmFAD2–1A* and *GmFAD2–1B*) into the same genetic background can effectively raise oleic acid content up to 80%, by disrupting the desaturase pathway (Tang et al., 2005; Pham et al., 2010). Crispr-Cas9 successfully produced double homozygous mutant plants showing a high oleic acid phenotypes (83.9%) compared to the wild-type soybean line (20.2%) (Do et al., 2019). Likewise, mutations in the three *FAD3* genes copies (*fad3a, fad3b, and fad3c*) have led to the development of ultra-low-linolenic soybean oil (less than 1%), positively affecting oil shelf life and reducing off-flavors (Hudson and Hudson, 2021). Additionally, combining mutations in *FAD3A* with mutations in *FAD2–1A* and *FAD2–1B* was proposed as a strategy to produce high oleic and low linolenic soybean oil (Demorest et al., 2016). Studies have demonstrated that developing soybean high-oleic varieties via *FAD* gene manipulation does not impact yield or protein production (Kim et al., 2015). This is pivotal because farmers still prioritise high-yielding and protein-rich varieties, which are the most valuable traits in the legume market.

It should be noted that both linoleic (ω6) and linolenic (ω3) acids are essential fatty acids for humans, and deficiencies in these fatty acids are linked to various health concerns (Choque et al., 2014). Hence, it could be posited that novel faba bean varieties may exhibit reduced nutritional value in comparison to conventional varieties with unmodified oil profiles. However, it is important to highlight that new varieties with reduced off-flavors should not entirely supplant traditional cultivars that have higher levels of linoleic and linolenic acids, as these varieties will only target a specific segment of the market. In addition, it is also crucial to acknowledge that faba bean is not an oil crop or a conventional source of polyunsaturated fatty acids (PUFAs). Therefore, regardless of the release of new reduced-flavor varieties on the market, it is imperative to obtain PUFAs from alternative sources to ensure a well-balanced and nutritious diet.

### 3.2 Targeting polyphenols and saponins to reduce bitterness and astringency

#### 3.2.1 Condensed tannins content is regulated by the *zt1* and *zt2* loci, but other candidate genes can be proposed to reduce their bitter precursors

Plant breeding efforts have led to the development of several low-tannin or zero-tannin faba bean varieties that are currently sold on the market. Low tannin genotypes present a reduction in condensed tannin content from ~8% to up to 0.01% on the dry matter (Oomah et al., 2011; Subodh Kumar and Amresh, 2018). Breeding for condensed tannins reduction mainly aimed at improving the nutritional value of animal feed (Guevara Oquendo et al., 2022).

There is a pleiotropic effect between tannin content in seeds and anthocyanic pigmentation on the flowers, which has facilitated genetic selection (Filippetti and Ricciardi, 1993). Condensed tannins and anthocyanins are, in fact, both end-products of the flavonoid pathway and they share metabolic routes. Anthocyanins are coloured pigments causing distinct colours in faba bean flowers. The wild-type flower is distinguished by its white petals, which feature a pronounced black spot on each wing petal and dark striae on the standard petal. Various color variations have been noted in the faba bean flowers, including an entirely white version, yellow wing spots, solid brown, pink, diffused yellow, red, etc. (Hughes et al., 2020). The absence of condensed tannins and presence of full white flowers share a simultaneous monogenetic control of two complementary and recessive genes, *zt1* and *zt2* (Jaakola et al., 2002; Gutierrez and Torres, 2019). Candidate genes for zt1 and zt2, which lead to a defect in the synthesis of anthocyanins or their precursors, have been proposed. The *Medicago* ortholog WD40 transcription factor TTG1 (Transparent Testa Glabra 1) was identified as the causal gene for the white flower phenotype (*zt-1* locus) (Webb et al., 2016), while the helix-loop-helix (bHLH) transcription factor TRANSPARENT TESTA8 (TT8) has been suggested as the candidate for *zt2* locus (Gutierrez et al., 2020). Despite TTG- and bHLH-conserved domain transcription factors being known to modulate flavonoid biosynthesis (Broun, 2005), their specific role in zero-tannin genotypes has not been elucidated. However, genomic breeding tools are available to differentiate two different mutant alleles for *TTG1*, *ttg1-a*, probably caused by a mutation in the promoter region, and *ttg1-b* due to a deletion at the 5′end of *VfTTG1*. Additionally, an allele-specific diagnostic marker has been developed to distinguish *zt-1* from both wild and *zt-2* genotypes (Gutierrez and Torres, 2019). These tools allow for marker assisted selection (MAS) for the zero-tannin trait.

**Figure 3:**
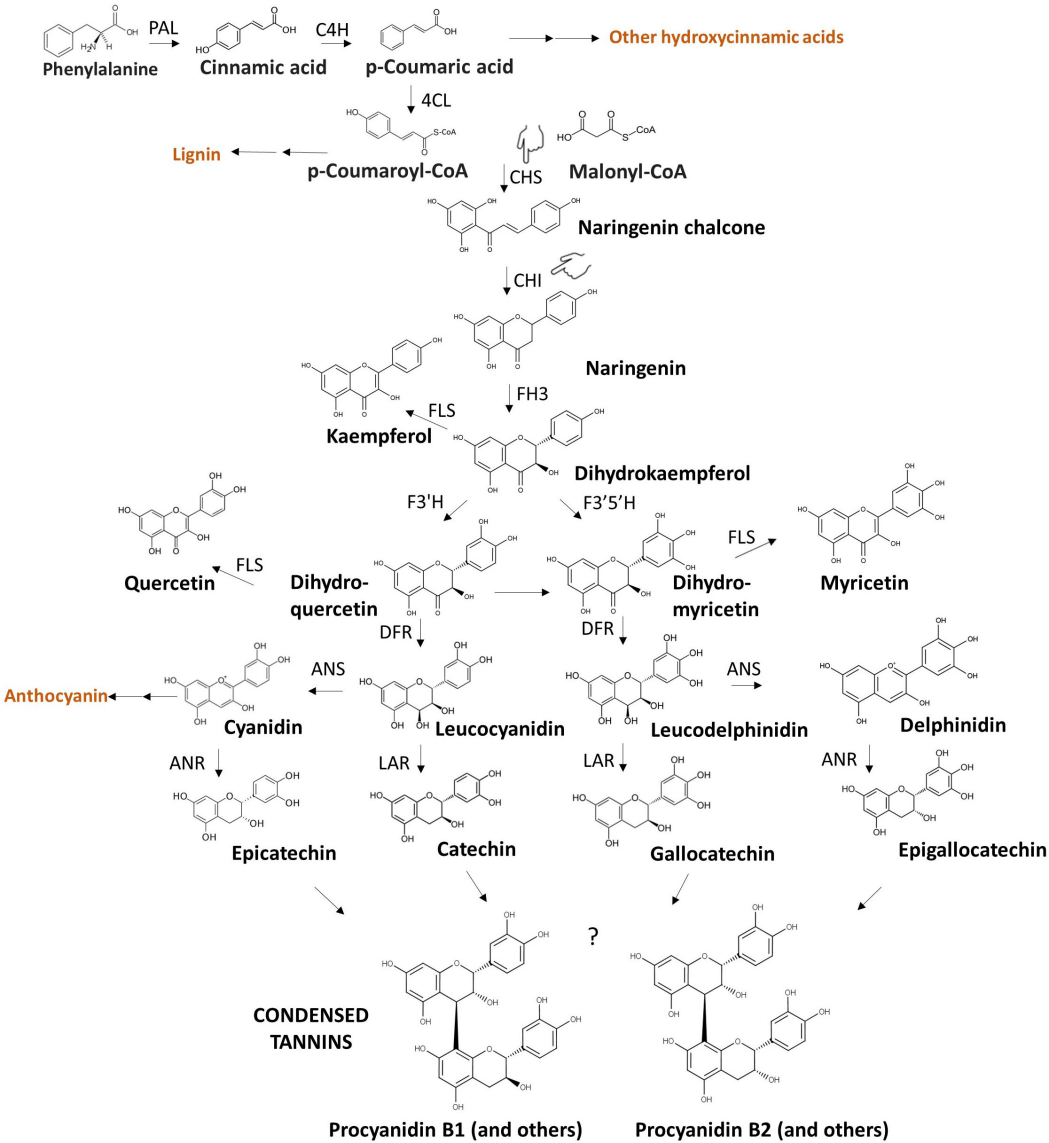
Condensed tannin biosynthesis. The illustration presents a streamlined version of the phenylpropanoid biosynthetic route involved in condensed tannin synthesis, resulting in various bitter and astringent polyphenols. Key intermediate compounds hereby represented belong to flavanone (naringenin), flavonol (quercetin, myricetin, kaempferol), leucoanthocyanidin (leucodelphinidin, leucocyanidin), anthocyanidin (delphinidin, cyanidin), flavan-3-ol (catechin, epicatechin, gallocatechin, epigallocatechin) classes. Compounds in orange are examples of important molecules that share precursors with condensed tannins. The acronym of each key enzyme is reported along the pathway: PAL, Phenylalanine ammonia lyase; CH4, cinnamate 4-hydroxylase; 4CL, 4-coumaroyl-CoA ligase; CHS, chalcone synthase; CHI, chalcone isomerase; F3H, flavanone-3-hydroxylase; F3’H, flavanone-3’-hydroxylase; F3’5’H, flavonoid-3’,5’-hydroxylase; ANS, anthocyanidins synthase; ANR, anthocyanidin reductase; LAR, leucoanthocyanidin reductase. The question mark in the last step of the pathways indicates that molecular mechanisms underlying the polymerization of flavan-3-ols into condensed tannins have not yet been elucidated. Procyanidin B1 and Procyanidin B2 are example of dimers belonging to the class of condensed tannins. The figure highlights the genes encoding CHI and CHS enzymes (pointing hand) as potential target genes for breeding applications to disrupt a portion of the phenylpropanoid biosynthetic pathway. *CHI* and *CHS* represent proposed alternative genes to *zt1* and *zt2*. The question mark indicates that the exact genetic control of the polymerization of flavan-3-ols in condensed tannins is unknown.

The phenylpropanoid biosynthetic pathway that leads to the synthesis of condensed tannins (depicted in **Figure 3**) is particularly complex and influenced by several genes and environmental factors (He et al., 2008). Various branches of this pathway yield bitter and/or astringent compounds (i.e., coumaric, caffeic, ferulic acid, myricetin, quercetin, etc.), therefore they are an interesting target for breeding for eliminating multiple compounds simultaneously. Along the pathways, L-phenylalanine, synthetised via the shikimate pathway, is converted into p-coumaroyl-Coa through a three-step reaction involving phenylalanine ammonia lyase (PAL), cinnamate 4-hydroxylase (CH4) and 4-coumaroyl-CoA ligase (4CL) (**Figure 3**). p-coumaric acids, an intermediate in subsequent reactions, can be a precursor of bitter phenolic acids such as caffeic, ferulic and sinapic acids, besides being converted to p-coumaroyl-Coa. Naringenin chalcone is synthesised from p-coumaroyl-CoA and malonyl-CoA via chalcone synthase (CHS), serving as the principal intermediate in flavonol biosynthesis. This compound undergoes catalysis by chalcone isomerase (CHI), transforming it into naringenin. Dihydroflavonols are subsequentially formed through sequential enzymatic actions involving flavanone-3-hydroxylase (F3H), flavanone-3’-hydroxylase (F3’H), and flavonoid-3’,5’-hydroxylase (F3’5’H), yielding dihydrokaempferol (DHK), dihydroquercetin (DHQ), and dihydromyricetin (DHM), respectively. Dihydroflavonols serve as the foundation for flavan-3-ols synthesis, which represents the core monomers in the complex condensed tannin structure (He et al., 2015; Rosa-Martinez et al., 2023). The enzymes anthocyanidins synthase (ANS), anthocyanidin reductase (ANR), and leucoanthocyanidin reductase (LAR) contribute to the formation of the flavan-3-ols catechin, gallocatechin, epicatechin, epigallocatechin from intermediates leucocyanidin and leucodelphinidin (Mora et al., 2022). Flavan-3-ols then undergo polymerization to create condensed tannins (proanthocyanidins), although the exact mechanism and genetic control remain still unknown. The enzymes codified by *zt1* and *zt2* are expected to act in the last steps of the pathway between the synthesis of dihydroflavonols and flavan-3-ols (Zanotto et al., 2020), modulating the pathway at the transcriptional level. In line with this hypothesis, Zanotto et al. (2020) found dihydroflavonols in the seeds of *zt1* and *zt2* genetic backgrounds, showing that zero tannins genotypes still contain the precursor molecules.

Despite breeding for reduced condensed tannin content being a successful story in faba bean, additional efforts are required to develop varieties with reduced off-flavor. A more precise understanding of specific tannin compounds and associated phenylpropanoid pathway genes is crucial. Potential interventions at the initial steps in the pathway could disrupt subsequent reactions that produce several taste-active molecules. Targeting genes such as *CHS* and *CHI* (marked by two pointing hands in **Figure 3**) could simultaneously reduce the level of flavonols kaempferol, quercetin, myricetin, and others, likely reducing bitterness. In order crops, the involvement of *chs*-like genes have been speculated. Novák et al. (2006) showed that a higher and more prolonged expression of chalcone synthase-like genes was found in maturing hop cones of cultivars with high bitterness compared to those in cultivars with a lower bitter profile. However, even in successful applications, some bitterness and/or astringency is expected to remain as disrupting *CHS* and *CHI* do not affect the synthesis of phenolic acids.

#### 3.2.2 Blocking the cyclisation of 2,3-oxydosqualene into saponins precursors as a strategy to mitigate off-taste and astringency

Amarowicz et al. (1997) isolated two distinct saponins possessing a chemical structure similar to that of soybean group B saponin in the seeds of four faba cultivars. The existence of B-type saponins (soyasaponin I/Bb) in faba bean was later verified, along with the detection of the DDMP saponin (soyasaponin βg)(Barakat et al., 2015). More recently, Stone et al. (2021) have detected and quantified the levels of the DDMP-soysaponins βa, αg and βg in faba bean seeds. Overall, the total saponin content in raw faba seeds ranges from approximately 481 to 757 µg/g, which is about half of the content detected in pea (1367 to 1701 µg/g) (Stone et al., 2021). The lower concentration of total saponins in *Vicia faba* compared to pea may be associated with a lower impact on taste. Recently, Tuccillo et al. (2022) corroborated the earlier findings of Heng et al. (2006) for pea, indicating that soyasaponin βg exhibits a greater degree of bitterness than soyasaponin Bb in faba bean ingredients. However, it has not been researched yet in detail whether saponins at their low content in faba bean impact the bitterness or astringency of faba bean products.

**Figure 4:**
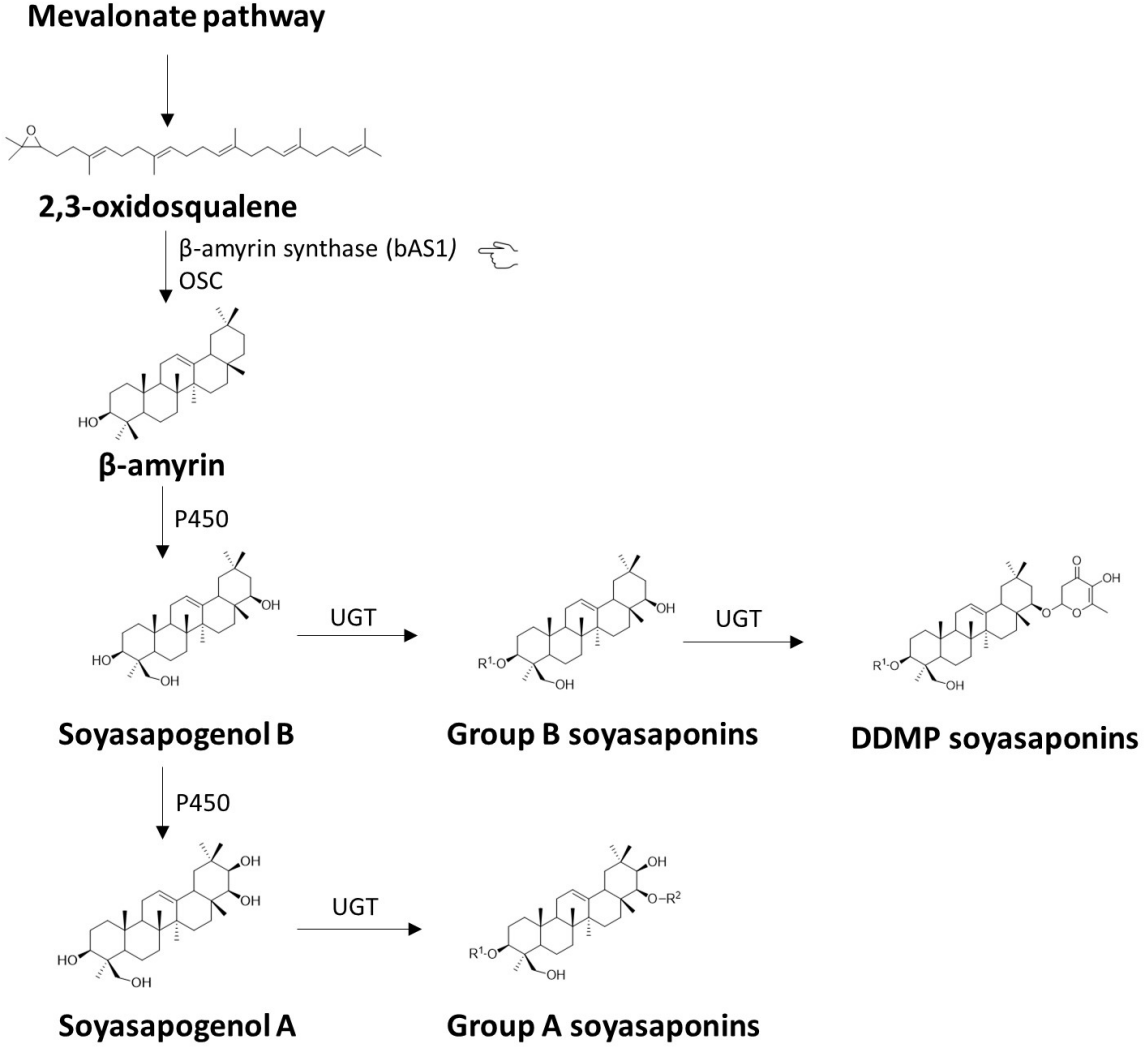
Biosynthesis of saponins. The figure illustrates a concise version of the proposed pathway for the synthesis of saponins in *Glycine max*. It is noteworthy to notice that saponin A reported in the pathway is present only in soybean, but not in faba bean. The synthesis of triterpenoid saponins begins with the cyclization of the precursor molecule 2,3-oxidosqualene into various triterpene scaffolds by the enzyme β-amyrin synthase (bAS), belonging to the family of oxidosqualene cyclases (OSCs). Among the produced scaffolds, β-amyrin is then subjected to site-specific oxidation reactions catalysed by cytochrome P450 monooxygenases (P450s), resulting in the formation of soyasapogenol B and soyasapogenol A. Subsequent glycosylation reactions catalysed by enzymes from the glycosyltransferase 1 superfamily, known as uridine diphosphate (UDP)-dependent glycosyltransferases (UGTs), produce structurally diverse triterpenoid saponins including, group B soyasaponin, DDMP-saponin and group A saponin. The highlighted β-amyrin synthase (*bAS*) gene (pointing hand) is a potential candidate for breeding applications because it plays a key role in the synthesis of saponins and is also located at a key point in the pathway.

Breeding for zero saponins content has been implemented in several crops, including legumes. In soybean, for instance, the biosynthetic pathway of saponins is well-studied (Chung et al., 2020; Sundaramoorthy et al., 2019b). A simplified version of this pathway is depicted in **Figure 4**. The 2,3-oxidosqualene is the key compound in triterpenoid saponin biosynthesis since the molecule is the common precursor for the different classes of saponins (Xu et al., 2004). The biosynthesis steps are known to begin with the cyclisation of the 2,3-oxidosqualene by a range of oxidosqualene cyclases (OSCs) into various triterpene precursors of saponins. Among them, β-amyrin synthase (bAS) is considered a primary OSC in plants and has been molecularly characterised in soybean (Chung et al., 2007; Mugford and Osbourn, 2013). The enzyme produces the aglycone β-amyrin, which is subsequently modified through a series of oxidations and sugar chain additions to form the final saponin compounds (Chung et al., 2007; Mugford and Osbourn, 2013). Previous studies in soybean and pea have demonstrated that reducing the saponin content in seeds is a feasible strategy. Takagi et al. (2011) successfully used RNA interference (RNAi) to silence the *bAS* gene and suppress saponin biosynthesis in soybean. Mutant lines of peas with the homozygous mutant *PsBAS1* (*b-amyrin synthase1*) gene accumulate virtually no saponins, paving the way for the development of peas with reduced bitterness and astringency (Vernoud et al., 2021). *β-amyrin synthase* genes are conserved in legumes as they have been cloned also in model legumes (Iturbe-Ormaetxe et al., 2003), suggesting that targeting *bAS* (indicated by the pointing hand in **Figure 4**) could be a viable approach for developing faba bean lines with reduced saponins and improved flavor.

## 4 Prospects for the development of faba bean ideotypes with neutral taste and aroma

Developing a breeding program to reduce off-flavors requires understanding the genetic basis of these traits and establishing breeding goals. Primary objectives involve reducing polyunsaturated fatty acid oxidation and polyphenol or saponin content in seeds, as schematised in **Table 2**. However, faba bean breeders must concurrently improve yield and stress resistance to ensure the crop’s success on the market. Faba bean is already well known to suffer yield instability, showing a large genotype by environment interaction (G × E) and lack of resilience to multiple abiotic and biotic stress conditions (Annicchiarico and Lannucci, 2008). Therefore, the inclusion of multiple traits in breeding schemes should be considered when designing breeding strategies. Selecting multiple traits will require understanding the genetic correlations between chemical attributes and agronomic performance (i.e., yield). Consequently, it will play a pivotal role to determine whether the genetic association between traits is favourable, unfavourable, or neutral and whether they are influenced by environmental components (Rana et al., 2019). Given that the biosynthesis of secondary metabolites is regulated by biotic and abiotic stress response (Thakur et al., 2019; Jan et al., 2021), it is expected that their concentration increase or decrease based on cultivation-specific conditions (Thakur et al., 2019). Oomah et al. (2014) have shown that the total extractable volatiles were highly dependent on the growing location of faba bean. Therefore, it will be crucial to assess the genotype-environment interaction for off-flavor related traits. Additionally, it is important to understand the heritability of these traits.

**Table 2:** Overview of breeding targets and candidate genes for off-flavor reduction in faba bean.

Trait	Candidate gene	Catalysed reaction	Expected outcome ^a^
Polyunsaturated fatty acids (PUFA)	*fatty acid desaturase 2 (fad2)*	Desaturation of oleic (C18:1) to form linoleic acid (C18:2)	Selecting *fad2* and *fad3* is expected to enhance oil stability by increasing the level of oleic acid and reducing the amounts of linoleic and linolenic acids (PUFA). Selecting for *lox* is expected to decrease PUFA oxidation, leading to a reduced release of volatile and non-volatile compounds. A diminished beany, grassy, earthy aroma is expected, as well as reduced bitterness due to lower presence of non-volatile lipid oxidation compounds.
	*fatty acid desaturase 3 (fad3)*	Desaturation of linoleic acid (C18:2) to form linolenic acid (C18:3)	
	*lipoxygenase (lox)*	Oxygenation of linoleic acid (C18:2) and linolenic acid (C18:3) to form hydroperoxides	
Condensed tannin	*zero tannin 1 (zt-1)*	Unknown exact function	Selecting *zt1* and *zt2* resulted in zero-tannin genotypes. It is anticipated that selecting *chs* and *chi* will lead to both reduced condensed tannin, such as procyanidins, and decreased precursors levels, respectively. Reduced bitterness and/or astringency is expected.
	*zero tannin 2 (zt-2)*	Unknown exact function	
	*chalcone synthase (chs)*	Synthesis of naringenin chalcone from 4-coumaroyl-CoA and three units of malonyl-CoA	
	*chalcone isomerase (chi)*	Isomerization of naringenin chalcone in naringenin	
Flavonoid	*chalcone synthase (chs)* *chalcone isomerase (chi)*	Synthesis of naringenin chalcone from 4-coumaroyl-CoA and three units of malonyl-CoA Isomerization of naringenin chalcone in naringenin	Selecting *chs* and *chi* is expected to disrupt the phenylpropanoid pathway, resulting in reduced levels of condensed tannin precursors, such as quercetin, myricetin, kaempferol, leucoanthocyanidins, anthocyanidins, and others. Reduced bitterness and/or astringency is expected.
Saponin	*β-amyrin synthase (bAS)*	Cyclization of 2,3-oxidosqualene to form β-amyrin	Selecting *bAS* is anticipated to disrupt the biosynthesis of saponins, consequently leading to a decrease in saponin B and DDMP saponin levels. A reduction in bitterness and/or astringency is expected as a result.
Vicine and convicine^b^	*Vicine and convicine 1 (vc1)*	Conversion of Guanosine-5’-triphosphate (GTP) into 2,5-diamino-6-ribosylamino-4(3*H*)-pyrimidinone 5′-phosphate (DARPP)^b^	Selecting *vc1* resulted in low vicine and convicine genotypes. It is expected that the full elimination of these molecules will be possible with the complete elucidation of the partially known biosynthetic pathway.

a The term “selecting” a particular gene refers to selecting a mutated version of the gene, which results in null or dysfunctional enzymes. The references supporting the statements in this summary table can be found in Section 3, where the pathways leading to the production of the molecules involved in off-flavors, specific genes, and potential gene manipulations are discussed.

b Vicine and convicine are reported in grey as their impact on taste has not been investigated, but they are expected to be bitter. See Bjornsdotter et al. (2021) for details on the molecular pathways controlling the vicine and convicine biosynthesis.

The table presents key breeding traits, candidate genes for selection in breeding programs, functions of enzymes encoded by these genes, and anticipated outcomes resulting from the selection of these genes.

In plant breeding, selection strategies are largely based on the crop’s reproduction system, which typically involves either self-pollination (where the same plant acts as both the male and female parent) or cross-pollination (where one plant serves as the male parent and another as the female parent). It is important to consider that faba bean demonstrates a mixed-mating system, exhibiting partial outcrossing (cross-pollination) behaviour with an average outcrossing rate of about 30%. The co-existence of selfing and outcrossing in the flowers of the same plant of faba bean requires modifications of traditional breeding methods for self-pollinated crops while also considering that strategies for highly outcrossing species may not be entirely applicable (Gnanasambandam et al., 2012). Flavour-neutral homozygous lines can be developed through line or family selection under insect-proof enclosures. These could be directly commercialised, adhering to the rules of distinctiveness, uniformity, and stability. However, high degrees of homozygosity in faba bean may result in a significant loss of heterosis, raising concerns from a yield perspective (Adhikari et al., 2021). In the absence of true F1 hybrids with maximised heterosis, synthetic varieties offer a viable option to partially exploit hybrid vigour (Brünjes and Link, 2021) and develop faba bean cultivars with reduced off-flavors.

To be able to identify the genetic components of off-flavors and to develop breeding tools in faba bean, there is the need to explore genetic variation. In this view, zero tannin germplasm can serve as a valuable starting material in breeding schemes, as it already has reduced bitterness and astringency. In addition to the zero-tannin germplasm, further investigation of natural variation in off-flavor-related traits within the faba bean germplasm is required. It is worth mentioning that the current genetic diversity of faba bean to exploit in breeding is restricted to the cultivated gene pool (Adhikari et al., 2021), as the wild ancestors of faba bean are officially considered still missing.

To optimise the utilization of the extensively available germplasm and identify potential lines for crossbreeding, creating smaller “core collections” may be beneficial, as these would be more accessible to breeders and easier to increase seed availability from selected accessions (Malosetti and Abadie, 2001; Khazaei et al., 2013). Since there is no existing evidence to select neutral-flavor candidates from genebanks, it is strategic to begin exploring accessions with diverse genetic backgrounds, breeding history, and adaptation to various latitudes. These factors are anticipated to impact metabolic chemical profiles, potentially resulting in a range of different flavor features (Lin et al., 2014; Kusano et al., 2015). Therefore, a core collection can be established by sampling materials based on genetic diversity, assuming that this diversity is reflected in flavor variation.

### 4.1 High-throughput screening methods are needed to identify flavor-neutral accessions within the large faba bean germplasm

Exploring over 43,000 accessions of faba bean preserved in approximately 40 gene banks (Duc et al., 2015) to identify rare flavor-neutral traits is challenging, time-consuming and costly. To perform in-house phenotyping, breeding companies must acquire machinery and techniques appropriate for screening volatile and non-volatile chemical compounds. The high cost of acquiring such equipment may deter some investigators, but collaborative research across various sectors could make these tools more accessible (Viana and English, 2021).

For volatile flavor compounds, Gas Chromatography (GC) in combination with mass spectrometry (MS) is a widely used analysis apparatus to separate a mixture of many volatile compounds to identify (and, if wanted, to quantify) each of the compounds. Before this analysis can take place, the volatiles first need to be extracted from faba bean flour. The extraction technique chosen determines the type and quantity of compounds extracted, as not all compounds can be extracted by each extraction method. Vacuum distillation and solvent extraction are accurate, but very laborious and time-consuming (Wang et al., 2021). Faster, but still relatively laborious is StirBar Sorptive Extraction (SBSE). Headspace solid-phase microextraction (HS-SPME) has emerged as the most frequently utilised technique for the analysis of volatile compounds from protein-rich plants (Wang et al., 2022). This technique requires minimal sample preparation and can be automated. It has been used to characterise several low- and high-tannin faba bean varieties (Oomah et al., 2014; Akkad et al., 2019). Moreover, SPME-GC-MS has also been employed in large screening in the context of soybean breeding (Ning et al., 2019; Wang et al., 2020). Despite its advantages of being fast, economical, and having high extraction efficiency, it has a medium throughput. A much shorter analysis time is taken using GC- Ion Mobility Spectrometry (IMS) (a few minutes). Another type of technique is Proton Transfer Reaction-Mass Spectrometry (PTR-MS), which is a high-throughput alternative method for detecting and quantifying volatile organic compounds in real-time (Acierno et al., 2020). Its non-destructive nature allows for fast analysis (about 1 minute per sample) and has been successfully used in apple and strawberry breeding (Zini et al., 2005; Carbone et al., 2006; Acierno et al., 2020). These techniques produce very large amount of data in a very short time. A disadvantage of these fast measurements is that the time needed to analyse the measured data is much higher than the time needed to perform the measurement itself. When data analysis is automated, GC-IMS or PTR-MS, could be rapid alternative phenotyping methods for breeding faba bean for improved flavors.

For non-volatile flavor compounds, high-performance liquid chromatography (HPLC) or ultra-high-performance liquid chromatography (UPLC) coupled with mass spectrometry (MS) are commonly used. These techniques can detect and quantify saponins, lipid derivatives, tannins, phenolics, and other compounds that influence taste perception and astringent mouthfeel. Extraction steps are crucial for analysing non-volatile compounds, as solvents used to impact the solubility of compounds (Wang et al., 2022). As for volatile compounds, also for non-volatile compounds applies that what and how much is detected depends on the extraction method used. Liquid chromatography–mass spectrometry was used to characterise phenolic profiles of seed coat and flower tissue of three faba bean genotypes. In this way, Zanotto et al. (2020) have determined the contents of flavonols, tannins, hydroxybenzoic and hydroxycinnamic acids among others, which are all compounds involved in taste perception. The composition of tannins in seed coats of faba beans was investigated by Merghem et al. (2004) using HPLC-MS. In order to increase throughput, UPLC is an alternative to HPLC. It offers improved method sensitivity, resolution, and speed (Swetha et al., 2020), making it a candidate analytical methodology for screening breeding trials. However, UPLC is still considered a laborious technology with medium throughput. Similarly laborious in the extraction, but much faster in the data analysis is the use of spectrometric determinations of total phenolics and of total tannins. However, these types of methods do not identify individual compounds and are not suitable for saponins and others. Near Infrared Spectroscopy (NIRS) provides a high-throughput alternative to traditional approaches for breeding applications. It is a light-based technology that predicts the chemical composition of seeds after developing a prediction model. Model development requires reference chemical data and absorbance values obtained after scanning samples under near-infrared light (Ozaki and Morisawa, 2021). NIR has already been adopted in faba bean to quantify the presence of tannins and total polyphenols and has the potential to be applied to other chemical compounds involved in off-flavors (Johnson et al., 2020), aiding in the fast development of improved faba bean varieties. Usually, NIR can predict chemical compounds as classes, but the quantification of individual compounds is usually hampered.

### 4.2 The availability of a reference genome opens a new scenario facilitating molecular and quantitative breeding

The field of faba bean genomics has historically trailed behind that of other legumes, resulting in a slower pace of advancements in trait improvement and breeding programs (Torres et al., 2011). Molecular markers and isozyme polymorphisms to assist faba bean breeding have been documented, but to a lesser extent than in other major crop species (Alghamdi et al., 2012). Several breeding traits were mapped using genetic maps constructed from bi-parental or multi-parental populations as extensively reviewed by Khazaei et al. (2021). However, except for the *zt1* and *zt2* loci involved in the biosynthetic pathways of tannins, there has been no attempt to map quantitative trait loci (QTL) or genes controlling off-flavors development in faba bean.

The lack of a historical focus on off-flavors in faba bean breeding presents a unique opportunity for modern genomics to contribute significantly to this field. The recent release of the giant genome sequence (~13 Gb) of the faba bean cultivar Hedin/2 (Jayakodi et al., 2023) opens a multitude of new possibilities for researchers and breeders (Jayakodi et al., 2023). Furthermore, a pan-genome initiative was launched by the University of Helsinki and Luke (Natural Resources Institute Finland) (Khazaei et al., 2021). This will further facilitate efficient utilization of genetic resources, and a more comprehensive understanding of genetic diversity within faba bean.

Mapping major genes and QTL associated with off-flavor development can expedite the discovery of novel genes and the identification of natural variation. The complexity of off-flavor pathways likely involves a combined effect of multiple alleles, requiring quantitative approaches in breeding. Several examples of quantitative approaches have been reported in legume breeding literature to map QTL regulating molecules with taste and aroma properties, including saponin, flavonoid, alkaloid, fatty acid compositions, and hexenal (Phan et al., 2007; Liang et al., 2010; Li et al., 2016; Li et al., 2017; Teraishi et al., 2017; Wang et al., 2020). The current low-cost high-density genotyping platforms can accelerate the selection process for these complex traits and enhance genome-wide association studies in faba bean (GWAS) (Pavan et al., 2020). GWAS enables fine mapping, but the construction of biparental or multi-parent advanced generation inter-cross (MAGIC) populations also offers a possibility for markers development within existing breeding programs (Khazaei et al., 2018). Due to the size of the faba bean genome, whole-genome resequencing for marker-assisted selection (MAS) is still cost-prohibitive. However, reduced representation sequencing approaches such as Genotyping by Sequencing (GBS), Restriction site-associated DNA sequencing (RADseq), and Double digest restriction-site-associated sequencing (ddRAD-seq) can be utilised for high-density scans of off-flavor related traits (Davey and Blaxter, 2010; Elshire et al., 2011; Truong et al., 2012). Additionally, the newly developed Single Primer Enrichment Technology (SPET) genotyping platform (commercialised as ALLEGRO) could serve as a valuable tool for dissecting the genetic architecture of off-flavors in faba bean (IGATech, 2022). SPET assay targets and types single nucleotide polymorphism (SNP) in 90,000 genic and intergenic loci across the faba bean genome, offering more flexibility than previous SNP arrays (Scaglione et al., 2019; Jayakodi et al., 2023).

Many candidate genes involved in the production of volatile and non-volatile compounds have been proposed in this review. Their functional role in the development of off-flavor in faba bean could be validated by CRISPR/Cas9 gene editing technology, which is revolutionising the research in plant breeding (Ahmad, 2023). CRISPR/Cas9-based genome editing employs the Cas9 endonuclease to make precise cuts in DNA. These cuts are guided by specific RNA sequences, known as gRNA, ensuring targeted modifications. Typically, CRISPR-CAS9 uses a transformation system, such as Agrobacterium-mediated transformation, which involves the introgression of foreign DNA into plant cells or tissues. Editing the genomes of most legumes presents challenges due to their transformation recalcitrance. As a consequence, alternative strategies for genome editing that bypass traditional transformation methods have been proposed and are currently evaluated (Nivya and Shah, 2023). Another limitation in applying genome editing to legumes is their low regeneration capacities (Baloglu et al., 2022). This refers to the ability of a genetically transformed plant cell or tissue to regenerate into a full plant that expresses and propagates the introduced traits. Despite the mentioned difficulties in applying genome editing to legume crops, example of successful transformations include soybean (Lu and Tian, 2022), *Lotus japonicus* (Wang et al., 2019*)*, *Medicago truncatula (* Jaudal et al., 2022*)*, cowpea (Bridgeland et al., 2023), peanut (Yuan et al., 2019), pea (Li et al., 2023), and chickpea (Gupta et al., 2023). To date, no CRISPR/Cas9 studies have been reported for faba bean (Bhowmik et al., 2021). However, the release of the reference genome aids in designing specific gRNAs to target specific genes. A tailored system for producing transgenic faba bean through Agrobacterium-mediated transformation exists (Hanafy et al., 2005), but there is a continuous need to refine protocols to overcome transformation and regeneration obstacles, which are also genotype-dependent.

In conclusion, the combination of newly available genomic resources, the development of mapping populations tailored to off-flavor traits, and the application of advanced genetic techniques such as CRISPR/Cas9 (which is currently primarily used for research purposes) hold great promise for accelerating progress in faba bean breeding. These advancements will ultimately contribute to the development of improved faba bean varieties that are manufactured into food products that are more appealing to consumers, helping to drive the expansion of meat and dairy analogues.

### 4.3 Potential drawbacks and considerations for breeding strategies to reduce off-flavors

Breeding to remove off-flavor compounds from faba bean seeds has the potential to improve the taste and palatability of meat or dairy analogues. However, this approach also carries some potential drawbacks. Most of the secondary metabolites likely involved in off-flavor (e.g., lipid oxidation products, flavonoids, tannins, saponins) are required by plants for various functions, such as pigmentation, growth, reproduction, and resistance to pathogens. As such, they represent adaptive traits that have undergone natural selection during evolution (Peter Constabel et al., 2014; Ku et al., 2022). The elimination of these secondary metabolites could cause a yield decrease.

Plants augment the production of polyphenols in response to abiotic stressors as a strategy to assist adaptation to drought, heavy metal exposure, salinity, extreme temperatures, and ultraviolet radiation (Sharma et al., 2019). Tannins, for example, play a role in frost protection, acting as supercooling-promoting agents or anti-ice nucleating agents (Koyama et al., 2014). Zero-tannin faba bean cultivars resulted in higher susceptibility to cold and frost damage of seeds. Faba bean is a crop at high risk for late-season frost injury in certain environments, which results in reduced yield and marketability (Inci and Toker, 2011; Henriquez et al., 2017).

Saponin represents a chemical barrier against pathogens (Zaynab et al., 2021). Positively, the disruption of the molecular pathway controlling the saponin biosynthesis in pea by mutational breeding has not shown negative effects on the physiological (germination capacity) or nutritional quality (protein content) of the seeds under controlled greenhouse conditions (Vernoud et al., 2021). However, field-based studies are needed to evaluate the agronomical performance of these plants under the pressure of pathogens, pests and, environmental cues.

Furthermore, removing secondary metabolites might negatively impact the nutritional value of the beans. For instance, some polyphenols serve as important sources of antioxidants and are considered health-promoting compounds (Ganesan and Xu, 2017). In addition, altering the composition of fatty acids results in seeds with diminished nutritional value. Any disruption of LOX pathways needs careful evaluation anyway, as these enzymes play a pivotal role in plant defense mechanisms. LOX mediates the biosynthesis of jasmonic acid upon wounding (Bell et al., 1995), but also contributes to the production of oxylipins, enhancing plant resistance against pathogens (Wilson et al., 2001).

Considering the potential drawbacks reported, it would be preferable in breeding to specifically target genes that are expressed within the seeds only. This allows the production of secondary metabolites in other plant tissues, ensuring the production of molecules that are involved in defense mechanisms against biotic or abiotic stresses in some parts of the plant. It is also important to account that the disruption of secondary metabolism may lead to unforeseen effects elsewhere in the plant’s metabolic system, which is complex and highly interconnected. The elimination of bitter and astringent compounds could change the availability of certain nutrients, which may lead to the production of new compounds or changes in the concentration of existing ones. Therefore, careful monitoring of any changes in the bean’s composition is crucial to ensure that no harmful or unwanted compounds are produced.

Establishing strong collaborations between plant breeders and the food industry, alongside rigorous monitoring and evaluation of the breeding program outcomes, will be crucial for ensuring that the development of new faba bean varieties aligns with both consumer preferences and the long-term sustainability of the crop.

## 5 Conclusions

The plant-based food industry is increasingly using faba bean as a key ingredient in meat and dairy alternatives. Although technological solutions can mitigate the off-flavors present in faba bean ingredients, they are energy-intensive and costly. We firmly believe that the most sustainable solution to eliminate off-flavors involves plant breeding, with a focus on developing varieties that are tailored for these applications and that ensure minimal to no off-flavors. Previous breeding research on other crops has identified genes behind off-flavor synthesis. Moreover, as we deepen our understanding of the molecules responsible for off-flavors, and with the onset of the genomic era in faba bean research, we can uncover additional genetic pathways. Such insights can significantly benefit faba bean breeding. The success of breeding initiatives aiming to improve off-flavor profiles depend on several factors, including the availability or creation of genetic variations, the quantitative nature of the trait, its heritability, and the efficiency of high-throughput screening methods, among others. For improved faba bean varieties with reduced off-flavors to be both competitive in the market and attractive to farmers, they must also exhibit high yield and resistance to both biotic and abiotic stresses. Complementing these traits in new varieties can promote the transition toward a more sustainable and climate-resilient diets.

## Author contributions

AL: Conceptualization, Formal Analysis, Visualization, Writing – original draft. WR: Formal Analysis, Investigation, Writing – review & editing. OB: Writing – review & editing. LP: Writing – review & editing, Funding acquisition, Project administration. LT: Funding acquisition, Project administration, Writing – review & editing, Conceptualization, Supervision.
